# Iron Intake and Human Health

**DOI:** 10.3390/nu16020206

**Published:** 2024-01-08

**Authors:** Gladys O. Latunde-Dada

**Affiliations:** Department of Nutritional Sciences, School of Life Course and Population Sciences, King’s College London, London SE1 9NH, UK; yemisi.latunde-dada@kcl.ac.uk

Iron deficiency anemia (IDA) is a global nutritional disorder affecting large population groups in varying magnitudes in different countries [1]. Such groups include vulnerable growing children, adolescent girls, pregnant and lactating women, and the aged. The physiological challenges of IDA, particularly those impacting growth and reproductive purposes, pose demands for increased daily iron requirements for these demographics. The daily iron needs of these vulnerable groups are commonly unmet because of inadequate iron intake, poor bioavailability, low absorption of iron due to the presence of inhibitors in the diet, or chronic blood loss, amongst others [2]. Consequently, evidence abounds regarding the deleterious and debilitating consequences of IDA on infants’ cognition, brain function, mental capability, work performance, and pregnancy outcomes [3,4]. Strategies to combat IDA include fortification of foods, iron supplementation to targeted groups, and attempts to explore and exploit food-based strategies to enhance iron absorption. For example, recent publications have revealed iron bioavailability data on novel food products, new oral iron supplements [5,6], and iron-biofortified foods that are safe and less toxic to the gut. Moreover, food processing procedures such as micro-milling and food encapsulation are employed to influence luminal bioaccessibility and iron absorption from foods [7]. Furthermore, gut microflora have emerged as important entities that could modify food matrices while secreting metabolites that might modulate iron absorption in the proximal gut region [8]. 

Besides the common causes of IDA, inflammation places a significant burden on systemic iron metabolism and the iron status of individuals with varying conditions. IDA in developing countries is often associated with inflammation and infections. Indeed, inflammation is a key regulator of hepcidin, the peptide hormone that modulates iron absorption and iron homeostasis [9]. Hepcidin expression is elevated in response to proinflammatory cytokines via a STAT3 transcriptional signaling pathway. This causes reduced iron absorption, since ferroportin, the iron efflux protein, is ubiquitinated by hepcidin [10]. Hence, acute or low-grade inflammation therefore contributes to the incidence of anemia due to the inhibition of iron absorption in the gut. In this issue, Htet [11] reported, in a randomized, double-blinded, placebo-controlled trial, the effect of sub-clinical inflammation (SCI) on the absorption of iron supplements in anemic adolescent schoolgirls in the Ayeyarwady region of Myanmar. The study also investigated the effect of combining vitamin A or folic acid with iron supplements given to the subjects with SCI. The study found that the efficiency of iron supplements, as expected, was inhibited by SCI and vitamin A, which mitigated their effect. Hence, the anti-inflammatory function of vitamin A neutralizes SCI to facilitate enhanced iron absorption. Moreover, previous studies have indicated the beneficial effect of vitamin A on the mobilization of stored iron for the enhancement of erythropoietin synthesis [12,13]. 

The prevalence of IDA is also associated with genetic variability in human populations [14]. For example, human genome-wide association studies (GWAS) have identified the association of multiple variants of the transmembrane protease serine 6 (TMPRSS6) enzyme with abnormal hematological biomarkers. As a negative modulator of hepcidin expression, TMPRSS6 down-regulates the transcription of hepcidin via the cleavage of hemojuvelin. Mutations in the TMPRSS6 gene lead to loss of function of the matriptase-2 protein, which induces increased hepcidin levels in the liver. Hence, elevated hepcidin levels, by degradation of ferroportin, lead to severe microcytic anemia due to reduced iron absorption in the gastrointestinal tract [15]. Shinta et al. [16] compared the association of two common TMPRSS6 SNPs (rs855791 and rs4820268) with iron intake and its effects on the prevalence of IDA in children under two years of age. While the two TMPRSS6 SNP variants were associated with lower serum ferritin concentrations, the association of IDA with iron intake was significantly greater in these children [16]. In contrast, however, De Oliveira Mota et al. [17] reported in the current issue that a red meat intake of 100 g/d would theoretically not reduce the estimated burden of IDA, particularly in menstruating females in population groups from France. This finding was obtained from a predictive mathematical model that quantitatively determined the health burden of IDA in terms of disability-adjusted life years (DALY) based on the age and gender of the subjects as well as their iron requirements, intake, and status in France. In another study in this issue [18], non-heme iron and total iron intake, rather than heme iron intake, were positively associated with MetS (waist circumference, reduced HDL-C, and elevated triglycerides and blood pressure) in the adult male population in China. An association between iron intake and MetS risk was reported only in men, but not in women, possibly because of reduced intake and increased loss of iron from the body due to the menstrual cycle in the latter. It should be noted, however, that the dietary iron intake was calculated from a 3-day diet record with inherent confounding issues. Nevertheless, evidence abounds in the literature [19,20,21] on the complex relationship between iron metabolism and the risk of MetS. The mechanism underlying these interactions is a subject of increasing research and may be linked to ferroptosis.

Moreover, the dysregulation of iron metabolism has been associated with the derangement of body tissue composition, energy expenditure, and metabolic syndrome. Moreno-Fernandez et al. [22] reported that Fe deficiency resulted in significant reductions in lean tissue mass and body fat, as well as inducing low energy expenditure in mice. While the levels of the triiodothyronine, thyroxine, and ghrelin hormones decreased in the anemic mice, glucose-dependent insulinotropic polypeptide (GIP), glucagon, insulin, corticosterone, and adrenocorticotropic hormone levels were elevated. Low oxygen consumption and hypoxia in the anemic mice may possibly have impacted the iron-dependent enzymes that are involved in ATP synthesis and the resultant weight loss in the mice, as was also reported by Schneider et al. [23].

The health burden of IDA also encompasses debilitating consequences on an infant’s growth, fatigue, and mental function. IDA in fetuses and babies during the pre- and post-natal periods could cause impaired memory, reduced attentiveness, and cognitive malfunction, amongst other issues [24]. Moreno-Fernandez et al. [25] performed a systematic literature review on the effect of iron status on the growth and developmental indices of premature infants. Iron demands are high during early growth, and it has been proposed to give iron supplements to vulnerable infants, particularly those with low birth weights. The study [24] also showed that low iron overload status in pre-term infants was more associated with lower birth weights than those with normal iron status. Hence, there is a delicate balance between the optimal iron supplementation dose to avert the negative consequences of IDA and the levels required to prevent organ damage due to iron overload. For example, a study [26] reported that some pregnant Norwegian women had iron supplementation beyond the daily recommended dose, which could cause adverse toxic effects to both the mothers and newborn babies. Nonetheless, a large-scale prospective study in the current issue revealed no deleterious consequences of iron supplementation administered during pregnancy and the postpartum period to the risk of developing Type 1 diabetes (T1D) in babies [27]. This Danish study concluded that maternal supplemental iron intake was possibly protective against T1D, rather than being a risk factor. However, there are instances when unabsorbed supplemented iron can induce the formation of toxic reactive oxygen species that cause inflammation or enhance the proliferation of iron-dependent pathogenic microbes in the distal gut region [28]. The deleterious consequence of high concentrations of supplemental iron in the colon has indeed been associated with gastrointestinal inflammation and microbial dysbiosis [29]. Iron chelation to starve and sequester iron from pathogenic microbes has been advocated as a panacea to maintain good gut health. Based on this premise, Horniblow et al. [30] investigated the safety and tolerability of a biopolymeric alginate as an iron chelator on hematological biomarkers and microbiome composition in healthy subjects. While the biopolymeric compound was safe and well tolerated, there were no changes to microbiota populations, and the iron-chelating ability of alginate was presumed to be confounded, possibly by the presence of other dietary chelators in the luminal food matrix content. Further published research by the same authors [31] showed that another biopolymer compound, namely, lignin, chelated iron and restricted its availability to detrimental proteobacteria while promoting the growth of favored beneficial bacteroides. Hence, lignin could be used to control microbial dysbiosis and inflammation associated with gut disorders. Regarding the public health significance of IDA, strategies to alleviate its effects include food fortification programs that are adopted by different countries and iron supplementation to specific vulnerable groups [32,33] across different age groups. Iron compounds used for supplementation include ferrous sulfate, elemental iron, ferric pyrophosphate, ferrous sulfate, ferric pyrophosphate, ferric ammonium citrate, ferrous sulfate, ferric pyrophosphate, fumarate, gluconate, and ferric ammonium citrate, among several others. Nevertheless, some of these iron salts cause gastrointestinal side effects such as nausea, constipation, diarrhea, and inflammation, as discussed earlier. Therefore, the initiative to synthesize novel iron salts that are safe, tolerable, and of high bioavailability is an ongoing challenge. Recently, newly synthesized nanoparticulate, ligand-modified Fe(III) polyoxo-hydroxide [34], nanoparticulate Fe pyrophosphates [35], sucrosomal iron [5], and iron multi-amino acid chelate (IMAAC) [6] have been added to the list of iron supplements to be used to prevent and treat IDA. In line with this development, Naviglio et al. [36] reported a method by which an iron (II) citrate complex was synthesized using iron filings and citric acid. The compound, FeC_6_H_6_O_7_∙H_2_O, which contains Fe(II), was purified with an array of analytical techniques, and it has been proposed to be available as an iron supplement for commercial purposes. For these reasons, it is imperative to evaluate and compare the in vitro bioaccessibility and in vivo bioavailability of FeC_6_H_6_O_7_∙H2O with the gold-standard FeSO_4_. Fe(II) is the form of iron that is absorbed by divalent metal transporter 1, DMT1, the metal transporter in the duodenum of the gastrointestinal tract. Yu et al. [37] investigated the transport of iron from ferrous bis-glycinate (Fe-Gly) in CRISPR-Cas 9 DMT1-knockout Caco-2 cells. These authors reported that iron from Fe-Gly was mainly absorbed by Caco-2 cells via DMT1, and that iron-regulated transporter (IRT)-like protein 14 (Zip14) expression was induced to compensate for iron absorption when DMT1 was limiting.

In summary, the papers in this issue, which were published originally in *Nutrients*, explored the significance of IDA, spanning across its causes and consequences. Furthermore, some approaches and interventions to prevent its incidence in human populations were highlighted and discussed. The health burden of IDA includes debilitating effects on energy metabolism, inflammation, and infection. Strategies to synthesize iron supplements of high bioavailability, with high redox inert and without adverse effects in the gastrointestinal tract, will be of immense benefit to the drive to prevent, manage, and treat IDA globally.

## References

[1] World Health Organization (2014). Micronutrient Deficiencies—Iron Deficiency Anaemia. http://www.who.int/nutrition/topics/ida/en/#.

[2] Aspuru K., Villa C., Bermejo F., Herrero P., García López S. (2011). Optimal management of iron deficiency anemia due to poor dietary intake. Int. J. Gen. Med..

[3] Munoz P., Humeres A. (2012). Iron deficiency on neuronal function. Biometals.

[4] Rufer E.S., Tran T.D., Attridge M.M., Andrzejewski M.E., Flentke G.R., Smith S.M. (2012). Adequacy of maternal iron status protects against behavioral, neuroanatomical, and growth deficits in fetal alcohol spectrum disorders. PLoS ONE.

[5] Elli L., Ferretti F., Branchi F., Tomba C., Lombardo V., Scricciolo A., Doneda L., Roncoroni L. (2018). Sucrosomial Iron Supplementation in Anemic Patients with Celiac Disease Not Tolerating Oral Ferrous Sulfate: A Prospective Study. Nutrients.

[6] Kajarabille N., Brown C., Cucliciu A., Thapaliya G., Latunde-Dada G.O. (2017). Bioavailability of iron multi-amino acid chelate preparation in mice and human duodenal HuTu 80 cells. Br. J. Nutr..

[7] Latunde-Dada G.O., Li X., Parodi A., Edwards C.H., Ellis P.R., Sharp P.A. (2014). Micromilling enhances iron bioaccessibility from wholegrain wheat. J. Agric. Food Chem..

[8] Kortman G.A.M., Reijnders D., Swinkels D.W. (2017). Oral iron supplementation: Potential implications for the gut microbiome and metabolome in patients with CKD. Hemodial Int..

[9] Silvestri L., Nai A., Dulja A., Pagani A. (2019). Hepcidin and the BMP-SMAD pathway: An unexpected liaison. Vitam. Horm..

[10] Nemeth E., Ganz T. (2021). Hepcidin-Ferroportin Interaction Controls Systemic Iron Homeostasis. Int. J. Mol. Sci..

[11] Htet M.K., Fahmida U., Dillon D., Akib A., Utomo B., Thurnham D.I. (2019). Is Iron Supplementation Influenced by Sub-Clinical Inflammation?: A Randomized Controlled Trial Among Adolescent Schoolgirls in Myanmar. Nutrients.

[12] Zimmermann M.B., Biebinger R., Rohner F., Dib A., Zeder C., Hurrell R.F., Chaouki N. (2006). Vitamin A supplementation in children with poor vitamin A and iron status increases erythropoietin and hemoglobin concentrations without changing total body iron. Am. J. Clin. Nutr..

[13] Semba R., Bloem M. (2002). The anemia of vitamin A deficiency: Epidemiology and pathogenesis. Eur. J. Clin. Nutr..

[14] Tanaka T., Roy C.N., Yao W., Matteini A., Semba R.D., Arking D., Walston J.D., Fried L.P., Singleton A., Guralnik J. (2010). A genome-wide association analysis of serum iron concentrations. Blood J..

[15] Gichohi-Wainaina W.N., Towers G.W., Swinkels D.W., Zimmermann M.B., Feskens E.J., Melse-Boonstra A. (2014). Inter-ethnic differences in genetic variants within the transmembrane protease, serine 6 (TMPRSS6) gene associated with iron status indicators: A systematic review with meta-analyses. Genes Nutr..

[16] Shinta D., Asmarinah, Adhiyanto C., Htet M.K., Fahmida U. (2019). The Association of TMPRSS6 Gene Polymorphism and Iron Intake with Iron Status among Under-Two-Year-Old Children in Lombok, Indonesia. Nutrients.

[17] Mota J.O., Tounian P., Guillou S., Pierre F., Membré J.M. (2019). Estimation of the Burden of Iron Deficiency Anemia in France from Iron Intake: Methodological Approach. Nutrients.

[18] Zhu Z., Wu F., Lu Y., Wu C., Wang Z., Zang J., Guo C., Jia X., Yao J., Peng H. (2018). Total and Nonheme Dietary Iron Intake Is Associated with Metabolic Syndrome and Its Components in Chinese Men and Women. Nutrients.

[19] Li Y., Zhao L., Yu D., Wang Z., Ding G. (2018). Metabolic syndrome prevalence and its risk factors among adults in China: A nationally representative cross-sectional study. PLoS ONE.

[20] Kilani N., Vollenweider P., Waeber G., Marques-Vidal P. (2015). Iron metabolism and incidence of metabolic syndrome. Nutr. Metab. Cardiovasc. Dis..

[21] Dos Santos Vieira D., Hermes Sales C., Galvão Cesar C., Marchioni D., Fisberg R. (2018). Influence of Haem, Non-Haem, and Total Iron Intake on Metabolic Syndrome and Its Components: A Population-Based Study. Nutrients.

[22] Moreno-Fernandez J., Díaz-Castro J., Alférez M.J.M., López-Aliaga I. (2019). Iron Deficiency and Neuroendocrine Regulators of Basal Metabolism, Body Composition and Energy Expenditure in Rats. Nutrients.

[23] Schneider J.M., Fujii M.L., Lamp C.L., Lönnerdal B., Dewey K.G. (2008). The use of multiple logistic regression to identify risk factors associated with anemia and iron deficiency in a convenience sample of 12–36-mo-old children from low-income families. Am. J. Clin. Nutr..

[24] Moreno-Fernandez J., Ochoa J.J., Latunde-Dada G.O., Diaz-Castro J. (2019). Iron Deficiency and Iron Homeostasis in Low Birth Weight Preterm Infants: A Systematic Review. Nutrients.

[25] Reid B.M., Georgieff M.K. (2023). The Interaction between Psychological Stress and Iron Status on Early-Life. Nutrients.

[26] Thorsen S.U., Halldorsson T.I., Bjerregaard A.A., Olsen S.F., Svensson J. (2019). Maternal and Early Life Iron Intake and Risk of Childhood Type 1 Diabetes: A Danish Case-Cohort Study. Nutrients.

[27] Størdal K., McArdle H.J., Hayes H., Tapia G., Viken M.K., Lund-Blix N.A., Haugen M., Joner G., Skrivarhaug T., Mårild K. (2018). Prenatal iron exposure and childhood type 1 diabetes. Sci. Rep..

[28] Mahalhal A., Williams J.M., Johnson S., Ellaby N., Duckworth C.A., Burkitt M.D., Liu X., Hold G.L., Campbell B.J., Pritchard D.M. (2018). Oral iron exacerbates colitis and influences the intestinal microbiome. PLoS ONE.

[29] Tang M., Frank D.N., Hendricks A.E., Ir D., Esamai F., Liechty E., Hambidge K.M., Krebs N.F. (2017). Iron in micronutrient powder promotes an unfavorable gut microbiota in Kenyan infants. Nutrients.

[30] Horniblow R.D., Mistry P., Quraishi M.N., Beggs A.D., Van de Wiele T., Iqbal T.H., Tselepis C. (2019). The Safety and Tolerability of a Potential Alginate-Based Iron Chelator; Results of A Healthy Participant Study. Nutrients.

[31] Horniblow R.D., Pathak P., Eshrati M., Latunde-Dada G.O., Tselepis C. (2023). Intestinal iron bio-accessibility changes by Lignin and the subsequent impact on cell metabolism and intestinal microbiome communities. Food Funct..

[32] Gera T., Sachdev H.S., Boy E. (2012). Effect of iron-fortified foods on hematologic and biological outcomes: Systematic review of randomized controlled trials. Am. J. Clin. Nutr..

[33] Tiwari A.K., Mahdi A.A., Chandyan S., Zahra F., Godbole M.M., Jaiswar S.P., Srivastava V.K., Negi M.P.S. (2011). Oral iron supplementation leads to oxidative imbalance in anemic women: A prospective study. Clin. Nutr..

[34] Hilty F.M., Arnold M., Hilbe M., Teleki A., Knijnenburg J.T.N., Ehrensperger F., Hurrell R.F., Pratsinis S.E., Langhans W., Zimmermann M.B. (2010). Iron from nanocompounds containing iron and zinc is highly bioavailable in rats without tissue accumulation. Nat. Nanotechnol..

[35] Pereira D.I.A., Bruggraber S.F.A., Faria N., Poots L.K., Tagmount M.A., Aslam M.F., Frazer D.M., Vulpe C.D., Anderson G.J., Powell J.J. (2014). Nanoparticulate iron(III) oxo-hydroxide delivers safe iron that is well absorbed and utilised in humans. Nanomedicine.

[36] Naviglio D., Salvatore M.M., Limatola M., Langella C., Faralli S., Ciaravolo M., Andolfi A., Salvatore F., Gallo M. (2018). Iron (II) Citrate Complex as a Food Supplement: Synthesis, Characterization and Complex Stability. Nutrients.

[37] Yu X., Chen L., Ding H., Zhao Y., Feng J. (2019). Iron Transport from Ferrous Bisglycinate and Ferrous Sulfate in DMT1-Knockout Human Intestinal Caco-2 Cells. Nutrients.

